# Personalizing age‐specific survival prediction and risk stratification in intracranial grade II/III ependymoma

**DOI:** 10.1002/cam4.2753

**Published:** 2019-12-03

**Authors:** Xiangyang Deng, Xiaojia Zhang, Liang Yang, Xiangqi Lu, Junhao Fang, Lisheng Yu, Dandong Li, Hansong Sheng, Bo Yin, Nu Zhang, Jian Lin

**Affiliations:** ^1^ Department of Neurosurgery The Second Affiliated Hospital and Yuying Children's Hospital of Wenzhou Medical University Wenzhou China

**Keywords:** grade II/III ependymoma, intracranial, nomogram, overall survival, SEER

## Abstract

**Background:**

Models for estimation of survival rates of patients with intracranial grade II/III ependymoma (EPN) are scarce. Considering the heterogeneity in prognostic factors between pediatric and adult patients, we aimed to develop age‐specific nomograms for predicting 3‐, 5‐, and 8‐year survival for these patients.

**Methods:**

A total of 1390 cases (667 children; 723 adults) of intracranial grade II/III EPNs diagnosed between 1988 and 2015 were extracted from the Surveillance, Epidemiology, and End Results (SEER) database for our study. Univariable and multivariable Cox analyses were employed to identify independent prognostic predictors. Age‐specific nomograms were developed based on the results of multivariate Cox analyses. We also evaluated the performance of these predictive models by concordance index, calibration curves, time‐dependent receiver operating characteristic curves, and decision curve analyses.

**Results:**

Considerable heterogeneity in prognostic factors was highlighted between pediatric and adult patients. Age, sex, tumor grade, surgery treatment and radiotherapy were identified as significant predictors of overall survival for children, and age, tumor grade, tumor size, surgery treatment, and marital status for adult. Based on these factors, age‐specific nomogram models were established and internally validated. These models exhibited favorable discrimination and calibration characteristics. Nomogram‐based risk classification systems were also constructed to facilitate risk stratification in EPNs for optimization of clinical management.

**Conclusions:**

We developed the first nomograms and corresponding risk classification systems for predicting survival in patients with intracranial grade II/III EPN. These easily used tools can assist oncologists in making accurate survival evaluation.

## Graphical abstract

Considerable heterogeneity in prognostic factors was highlighted between pediatric and adult patients with intracranial grade II/III ependymoma in our study. And the present study is, to our knowledge, the first study to develop nomograms for estimation of survival in ependymomas. We have demonstrated excellent discrimination and calibration of these predictive models. Besides, our nomogram models showed superiority in the clinical utility as well. Moreover, the novel proposed nomogram‐based risk classification systems can also help neurosurgeons to classify risk more objectively.

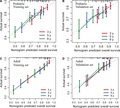

## 1 INTRODUCTION

Intracranial ependymomas (EPNs) constitute the second most prevalent malignant brain tumor in children, while only account for approximately 3%‐5% of central nervous system (CNS) tumors in adult.^1^, ^2^, ^3^ These tumors are thought to arise from ependymal cells lining the cerebral ventricles, spinal cord central canal, and cortical rests.^4^, ^5^ According to the World Health Organization (WHO) classification system,^6^ EPNs have traditionally been divided into 3 grades, grade I subependymomas and myxopapillary, grade II classic EPNs and grade III as anaplastic type. Clinical management of intracranial grade II/III EPN is usually challenging for its locally invasive growth pattern.^7^ For substantial heterogeneity in the natural course and prognosis of this disease, estimating survival is always difficult, even for experienced neurosurgeon. Recognizing the importance of survival prediction for assisting in clinical decision making and optimization of therapeutic approaches in clinical care, a precise prognostic tool tailored to individual patient factors is need for these patients.

At present, WHO grading is employed to stratify the EPNs from throughout the CNS for predicting survival outcomes.^7^ However, this WHO grade‐based risk classification is considered contentious and inconclusive for its limited predictive power without good consistent associations with patient prognosis.^7^, ^8^, ^9^, ^10^ To date, there is no reliable statistical prediction model designed to conduct individualized estimation of survival for patients with intracranial EPN.

In addition, despite the age‐ and site‐specific prognostic factors among these tumors, for its rarity, some studies grouped all ages and occurrence sites together for survival analysis, making the differences among subsets unclear. Thus, in this study, we sought to conduct comprehensive prognostic evaluations focusing on the primary intracranial grade II/III EPNs in children and adults, and develop nomograms for reliable estimation of 3‐, 5‐, and 8‐year survival.

## 2 METHODS

### 2.1 Study population

The data of study population were extracted from the National Cancer Institute's Surveillance, Epidemiology, and End Results (SEER) database in the United States. The SEER program is a population‐based cancer registry system capturing data on patient demographics, clinicopathologic features, and cancer‐associated treatment, covering nearly 30% of the US population.^11^ The inclusion criteria were as follows: (a) diagnosed with malignant EPN as defined by the International Classification of Diseases for Oncology Third Edition (ICD‐O‐3) histology codes 9391/3, 9392/3 and 9393/3 (ependymoma, anaplastic ependymoma, and papillary ependymoma); (b) diagnosed from 1988 to 2015; (c) primary tumor location labeled C71.0‐C71.9 according to ICD‐O‐3 site; (d) EPN was the only or the first malignancy; (e) patients had complete follow‐ups. And patients with unknown race, tumor extension, tumor size, surgery type, and radiotherapy were excluded.

### 2.2 Study variables and endpoints

Following variables were extracted from the SEER database including age at diagnosis, race (white or non‐white), sex (male or female), histological type (grade II: classic EPN; grade III: anaplastic EPN), tumor location (supratentorial, infratentorial, or others), tumor size, tumor extension, surgery treatment, radiotherapy (yes or no), chemotherapy (yes or no), and marital status (married, single or unknown). For pediatric patients (<18 years), the age was divided into three groups, ≤2 years, 3‐7 years, and 8‐17 years, based on the best cut‐off points determined by X‐tile program (Figure S1A‐C). Similarly, the age of adult patients (>18 years) was grouped as 18‐53 years, 54‐68 years, and >68 years (Figure S1D‐F). Tumor size was grouped by median value in both pediatric and adult cohorts. Tumor extension was divided into localized, regional and distant, in accordance with our previous report.^12^ According to SEER site‐specific coding guidelines, the extent of surgical resection was categorized as no surgery, biopsy/subtotal resection (STR), and gross total resection (GTR). Overall survival (OS) was defined as the primary outcome.

### 2.3 Nomogram development and statistical analyses

In both pediatric and adult cohorts, patients were randomly divided into training and validation sets at a ratio of 7:3. Univariate and multivariate Cox proportional hazards regression analyses were conducted to identify prognostic factors (*P* < .05) that significantly associated with OS in the training groups. Ground on the results of multivariate Cox analyses, nomograms were established to estimate 3‐, 5‐, and 8‐year OS rates for EPN patients. Then, the nomogram models were internally validated. Discrimination ability of the nomograms were evaluated by concordance index (C‐index), and time‐dependent receiver operating characteristic curve (ROC) with the area under the curve (AUC) value. Calibration curve was applied to access the consistency between nomogram predicted OS and actual outcome. Bootstrap resampling (1000 resamples) were conducted for these analyses. Additionally, the clinical usefulness of the nomogram models was accessed by decision curve analysis (DCA).

In addition, we calculated the scores for each patient in the training cohorts using the nomogram models. Then, according to the total score of each patient, risk classification systems were established to assign patients into a high‐, intermediate‐, or low‐risk group by best cut‐off values. And Kaplan‐Meier curve and the log‐rank test were employed to illustrate and compare the OS of patients in the different risk groups.

The X‐tile program (version 3.6.1) was applied to determine the best cut‐off points. Survival curves were depicted using Kaplan‐Meier method. Comparisons across age categories and random groups used Chi‐square tests or Student *t* tests, as appropriate. The SEER*Stat software (version 8.3.5), SPSS software (version 24.0) and R software (version 3.13) were used for all statistical analyses. *P* value <.05 was considered statistically significant.

## 3 RESULTS

### 3.1 Patient characteristics

Owing to known differences in multiple characteristics between pediatric and adult patients with EPN, 1390 eligible patients were divided by age (667 children; 723 adults) for all analyses (Table 1). Overall, more than half (52.9%) of the patients were male, and white (80.4%) accounted for the vast majority population. Based on comparative analyses, pediatric and adult patients were fairly well balanced regarding to sex and race; however, statistically significant differences were found between two groups for tumor grade distribution, tumor site, tumor extension, tumor size and treatment options. Grade III EPN (42.9% vs 22.5%) and tumor with larger size (50.95 vs 37.69 mm) were more common observed in children, and these patients were more likely to receive GTR (60.6% vs 45.1%), radiotherapy (70.0% vs 58.4%) or chemotherapy (36.7% vs 5.9%) than adult patients. In addition, the baseline characteristics were comparable between the training and validation cohorts in both pediatric and adult cohorts, as summarized in Table 2.

**Table 1:** Patient characteristics in the study

Variable	Category	All	Pediatric	Adult	*P* a
Total		1390 (100%)	667 (100%)	723 (100%)	
Age	≤2	238 (17.1%)	238 (35.7%)		
	3‐7	218 (15.7%)	218 (32.7%)		
	8‐17	211 (15.2%)	211 (31.6%)		
	18‐53	486 (35.0%)		486 (67.2%)	
	54‐68	160 (11.5%)		160 (22.1%)	
	>68	77 (5.5%)		77 (10.7%)	
Sex	Female	654 (47.1%)	307 (46.0%)	347 (48.0%)	.463
	Male	736 (52.9%)	360 (54.0%)	376 (52.0%)	
Race	White	1117 (80.4%)	528 (79.2%)	589 (81.5%)	.280
	Non‐white	273 (19.6%)	139 (20.8%)	134 (18.5%)	
WHO grade	Grade II	941 (67.7%)	381 (57.1%)	560 (77.5%)	**<.001**
	Grade III	449 (32.3%)	286 (42.9%)	163 (22.5%)	
Tumor site	Supratentorial	590 (42.4%)	245 (36.7%)	345 (47.7%)	**<.001**
	Infratentorial	543 (39.1%)	256 (38.4%)	287 (39.7%)	
	Others	257 (18.5%)	166 (24.9%)	91 (12.6%)	
Tumor extension	Localized	1131 (81.4%)	519 (77.8%)	612 (84.6%)	**.001**
	Regional	236 (17.0%)	131 (19.6%)	105 (14.5%)	
	Distant	23 (1.7%)	17 (2.5%)	6 (0.8%)	
Tumor size	Mean (mm)	44.06	50.95	37.69	**<.001**
	Median (mm)	41	48	37	
Surgery	No surgery	80 (5.8%)	17 (2.5%)	63 (8.7%)	**<.001**
	Biopsy/STR	580 (41.7%)	246 (36.9%)	334 (46.2%)	
	GTR	730 (52.5%)	404 (60.6%)	326 (45.1%)	
Radiotherapy	No	501 (36.0%)	200 (30.0%)	301 (41.6%)	**<.001**
	Yes	889 (64.0%)	467 (70.0%)	422 (58.4%)	
Chemotherapy	No	1102 (79.3%)	422 (63.3%)	680 (94.1%)	**<.001**
	Yes	288 (20.7%)	245 (36.7%)	43 (5.9%)	
Marital status	Single			301 (41.6%)	
	Married			381 (52.7%)	
	Unknown			41 (5.7%)	

Note

The bold values represent statistical significance.

Abbreviations: GTR, gross total resection; STR, subtotal resection.

a Chi square test or Student *t* test.

**Table 2:** Patient characteristics of the trainings and internal validations

Variable	Category	Pediatric			Adult		
		Training	Validation	*P* a	Training	Validation	*P* a
Total		466 (100%)	201 (100%)		506 (100%)	217 (100%)	
Age	≤2	161 (34.5%)	77 (38.3%)	.635			
	3‐7	154 (33.0%)	64 (31.8%)				
	8‐17	151 (32.4%)	60 (29.9%)				
	18‐53				339 (67.0%)	147 (67.7%)	.777
	54‐68				115 (22.7%)	45 (20.7%)	
	>68				52 (10.3%)	25 (11.5%)	
Sex	Female	222 (47.6%)	85 (42.3%)	.203	246 (48.6%)	101 (46.5%)	.609
	Male	244 (52.4%)	116 (57.7%)		260 (51.4%)	116 (53.5%)	
Race	White	362 (77.7%)	166 (82.6%)	.152	414 (81.8%)	175 (80.6%)	.710
	Non‐white	104 (22.3%)	35 (17.5%)		55 (18.2%)	42 (19.3%)	
WHO grade	Grade II	264 (56.7%)	117 (58.2%)	.709	392 (77.5%)	168 (77.4%)	.988
	Grade III	202 (43.3%)	84 (41.8%)		114 (22.5%)	49 (22.6%)	
Tumor site	Supratentorial	174 (37.3%)	71 (35.3%)	.701	241 (47.6%)	104 (47.9%)	.700
	Infratentorial	174 (37.3%)	82 (40.8%)		198 (39.1%)	89 (41.0%)	
	Others	118 (25.3%)	48 (23.9%)		67 (13.2%)	24 (11.1%)	
Tumor extension	Localized	367 (78.8%)	152 (75.6%)	.630	425 (84.0%)	187 (86.2%)	.623
	Regional	87 (18.7%)	44 (21.9%)		76 (15.0%)	29 (13.4%)	
	Distant	12 (2.6%)	5 (2.5%)		5 (1.0%)	1 (0.5%)	
Tumor size(mm)	<48	241 (51.7%)	95 (47.3%)	.291			
	≥48	225 (48.3%)	106 (52.7%)				
	<37				260 (51.4%)	101 (46.5%)	.233
	≥37				246 (48.6%)	116 (53.5%)	
Surgery	No surgery	14 (3.0%)	3 (1.5%)	.524	48 (9.5%)	15 (6.9%)	.411
	Biopsy/STR	171 (36.7%)	75 (37.3%)		236 (46.6%)	98 (45.2%)	
	GTR	281 (60.3%)	123 (61.2%)		222 (43.9%)	104 (47.9%)	
Radiotherapy	No	140 (30.0%)	60 (29.9%)	.960	207 (40.9%)	94 (43.3%)	.547
	Yes	326 (70.0%)	141 (70.1%)		299 (59.1%)	123 (56.7%)	
Chemotherapy	No	298 (63.9%)	124 (61.7%)	.579	480 (94.9%)	200 (92.2%)	.160
	Yes	168 (36.1%)	77 (38.3%)		26 (5.1%)	17 (7.8%)	
Marital status	Single				204 (40.3%)	97 (44.7%)	.268
	Married				276 (54.5%)	105 (48.4%)	
	Unknown				26 (5.1%)	15 (6.9%)	

Abbreviations: GTR, gross total resection; STR, subtotal resection.

a Chi square test or Student *t* test.

### 3.2 Independent prognostic predictors for pediatric and adult patients

**Figure 1:**
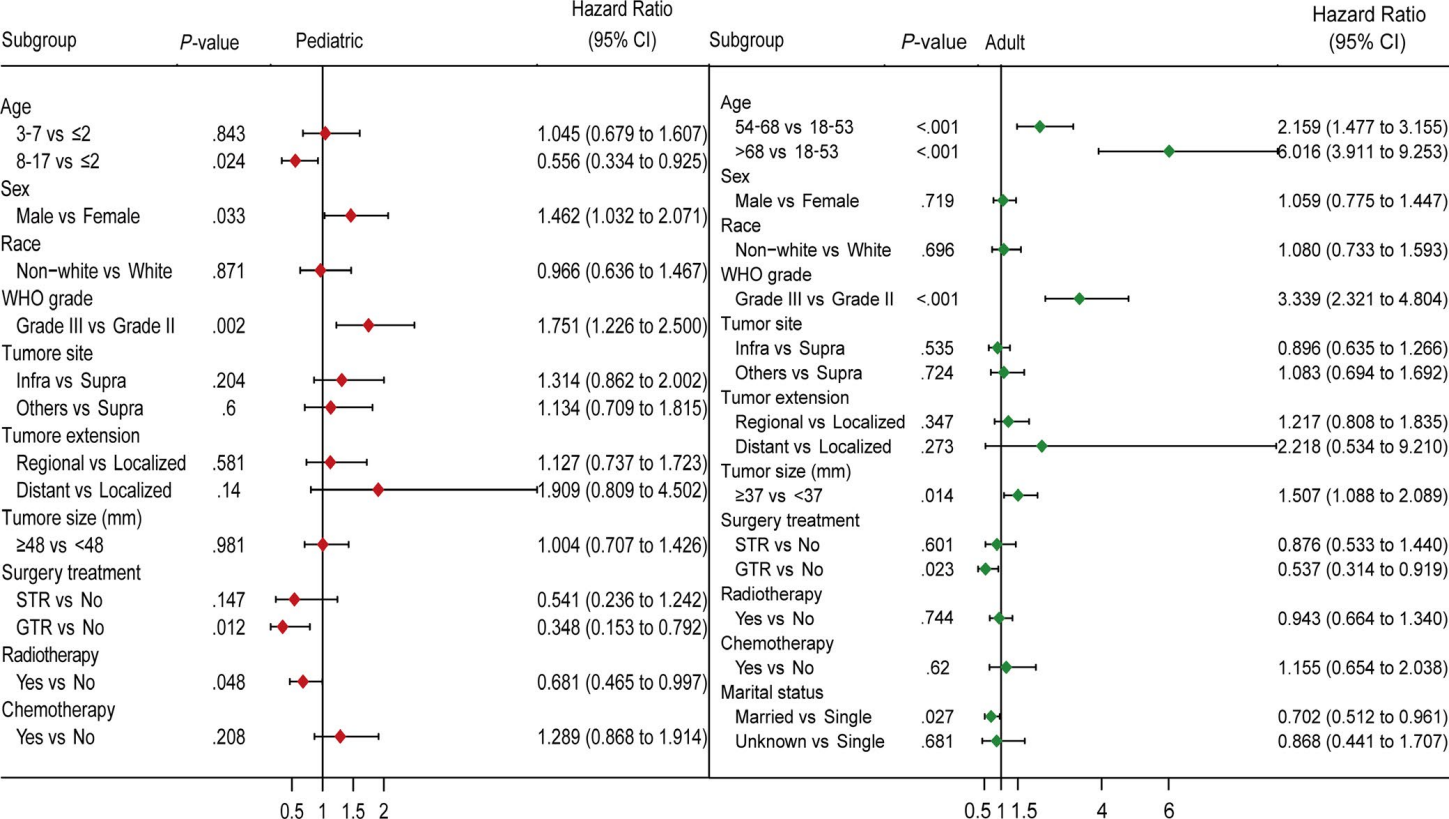
Forest plots showing results of multivariate Cox analyses in the training cohorts. GTR, gross total resection; Infra, infratentorial; Supra, supratentorial; STR, subtotal resection

In both pediatric and adult training cohorts, age, tumor grade, surgery treatment, and chemotherapy were significantly associated with OS using univariate Cox analyses (*P* < .05, Table 3). Radiation treatment was significantly associated with OS in children whereas tumor site and size were significantly associated with outcomes in adult. After controlling for confounding variables by multivariate Cox analyses, ultimately, age, sex, tumor grade, surgery treatment, and radiotherapy were identified as independent predictors of OS for pediatric patients, and age, tumor grade, tumor size, surgery treatment, and marital status for adult patients (Figure 1).

**Table 3:** Univariate Cox analyses in the training cohorts

Variable	Category	*P*	
		Pediatric	Adult
Age	3‐7 vs ≤2	.211	
	8‐17 vs ≤2	**<.001**	
	8‐17 vs 3‐7	**.005**	
	54‐68 vs 18‐53		**.003**
	>68 vs 18‐53		**<.001**
	>68 vs 54‐68		**<.001**
Sex	Male vs Female	.073	.132
Race	Non‐white vs White	.745	.566
WHO grade	Grade III vs Grade II	**.009**	**<.001**
Tumor site	Infra vs Supra	.111	**.026**
	Others vs Supra	.183	.783
	Others vs Infra	.910	.065
Tumor extension	Regional vs Localized	.165	.398
	Distant vs Localized	.070	.582
	Distant vs Regional	.291	.756
Tumor size(mm)	≥48 vs <48	.889	
	≥37 vs <37		**.016**
Surgery	Biopsy/STR vs No surgery	.100	.165
	GTR vs No surgery	**.004**	**<.001**
	GTR vs Biopsy/STR	**.006**	**<.001**
Radiotherapy	Yes vs No	**.008**	.909
Chemotherapy	Yes vs No	**.001**	**.002**
Marital status	Married vs Single		.081

Note

The bold values represent statistical significance.

Abbreviations: GTR, gross total resection; Infra, infratentorial; Supra, supratentorial; STR, subtotal resection.

### 3.3 Construction and validation of the age‐specific nomograms

**Figure 2:**
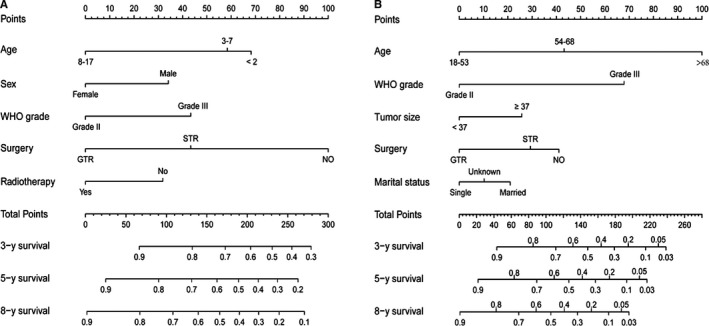
Nomograms estimating the probability of 3‐, 5‐ and 8‐year overall survival for pediatric (A) and adult (B) patients with intracranial grade II/III ependymoma. STR, subtotal resection; GTR, gross total resection

**Figure 3:**
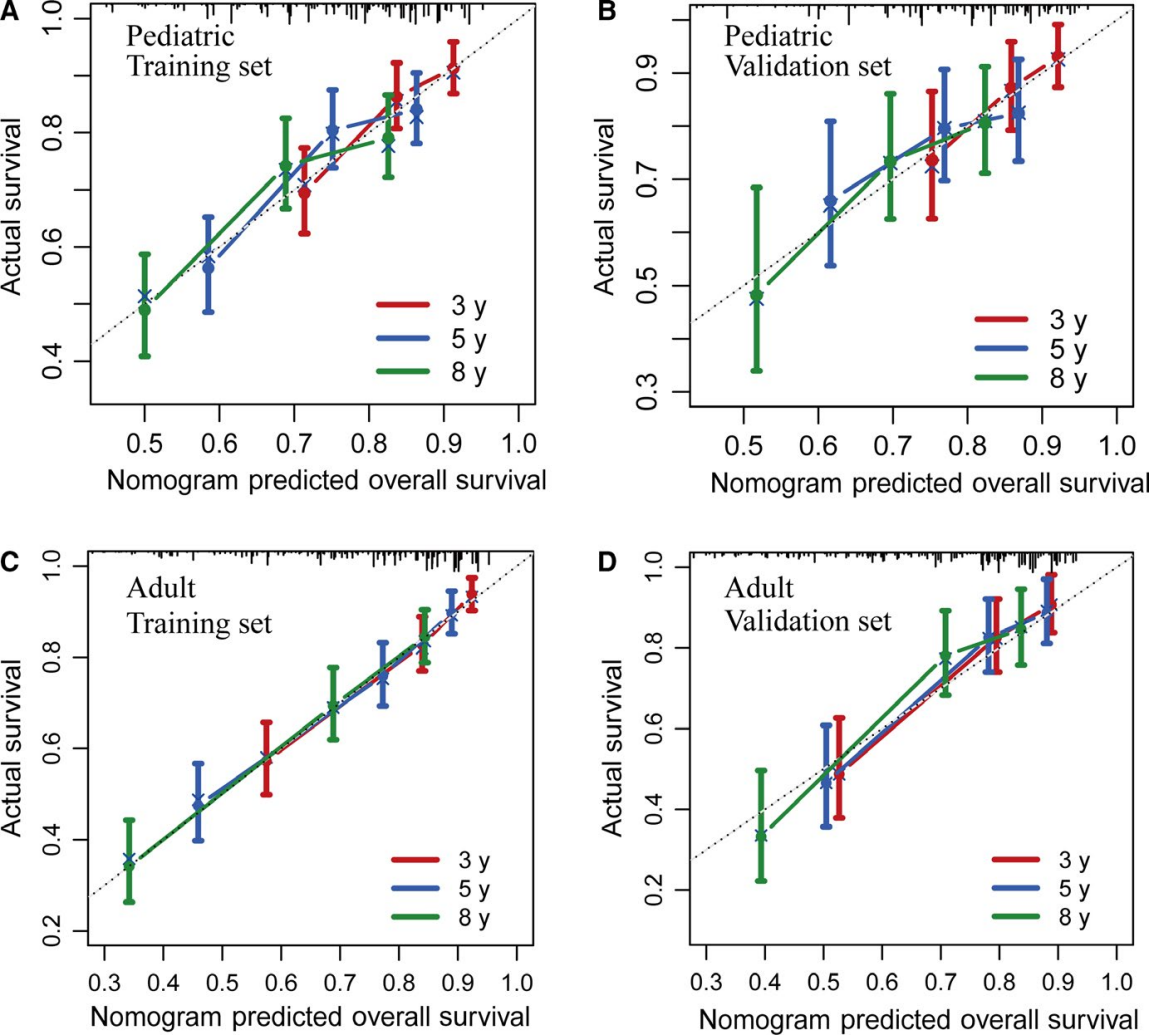
Calibration curves for predicting patient survival at each time point in the pediatric training set (A), pediatric validation set (B), adult training set (C), and adult validation set (D). Nomogram‐predicted survival is plotted on the x‐axis, and actual survival is plotted on the y‐axis. A plot along the 45‐degree line (dashed line) would indicate a perfect calibration model in which the predicted probabilities are identical to the actual outcomes

To individualize the 3‐, 5‐, and 8‐year predicted OS probability for pediatric and adult patients, nomograms were developed on basis of the results of multivariate Cox analyses, and were internally validated (Figure 2). The final age‐specific nomograms for children and adult showed favorable discrimination in the training cohorts (C‐index for pediatric, 0.67; C‐index for adult, 0.74), and the C‐index were similar in the validation cohorts (0.66 for pediatric and 0.74 for adult). With respect to calibration, excellent tracking of nomogram predictions vs observed outcomes was also observed (Figure 3).

**Figure 4:**
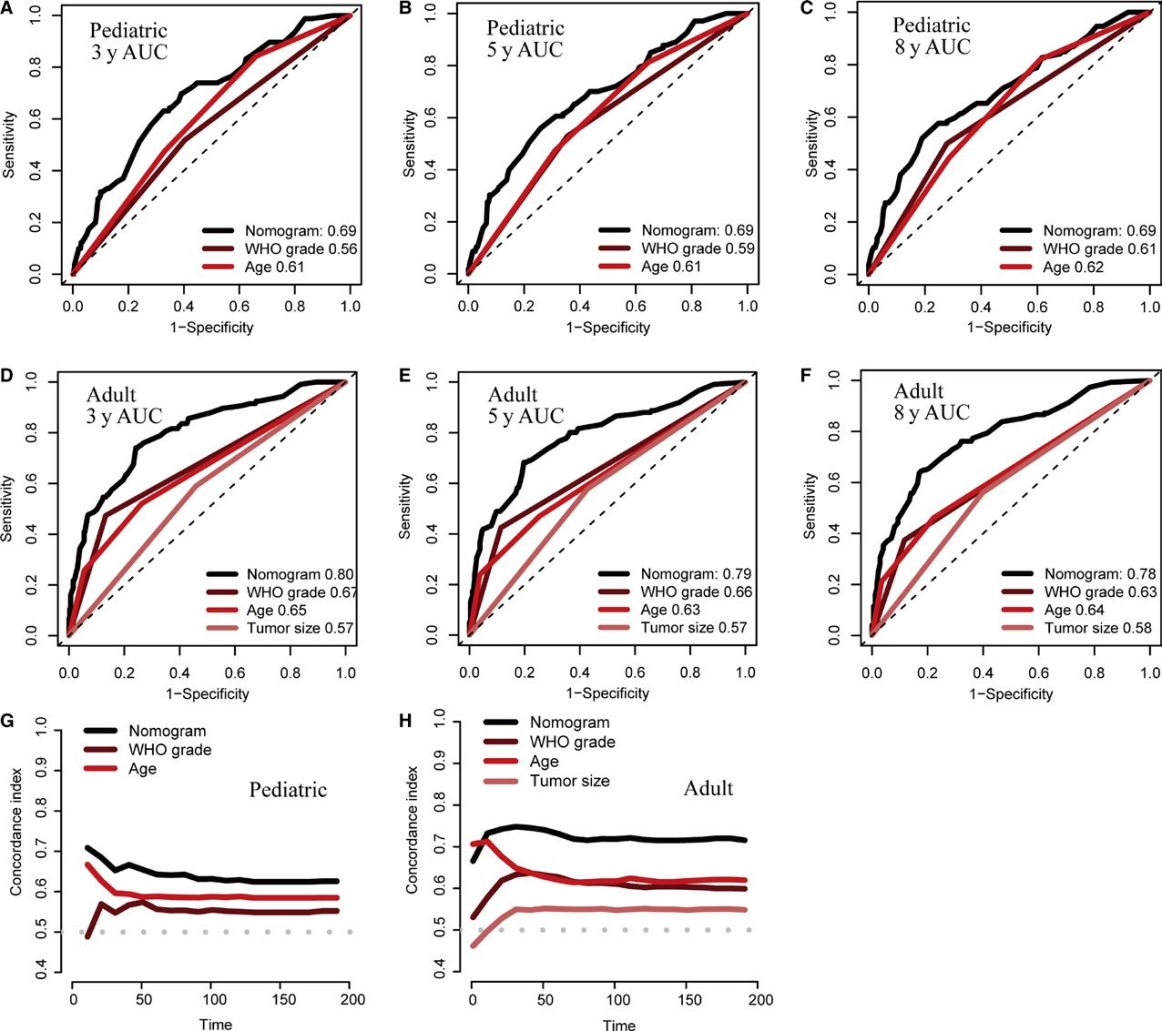
The prognostic performances were compared between nomogram models and different conventional clinical characteristics by ROC curves and time‐dependent C‐index. Comparison of the ROC curves of the nomogram model and different conventional clinical characteristics for 3‐ (A), 5‐ (B) and 8‐y (C) OS prediction in the pediatric training set, and 3‐ (D), 5‐ (E) and 8‐y (F) OS prediction in the adult training set; The prognostic performance was compared between the nomogram model and different conventional clinical characteristics by calculating the C‐index in the pediatric (G) and adult (H) training sets. AUC, areas under the ROC curve; C‐index, concordance index; ROC, receiver operating characteristic curve

**Figure 5:**
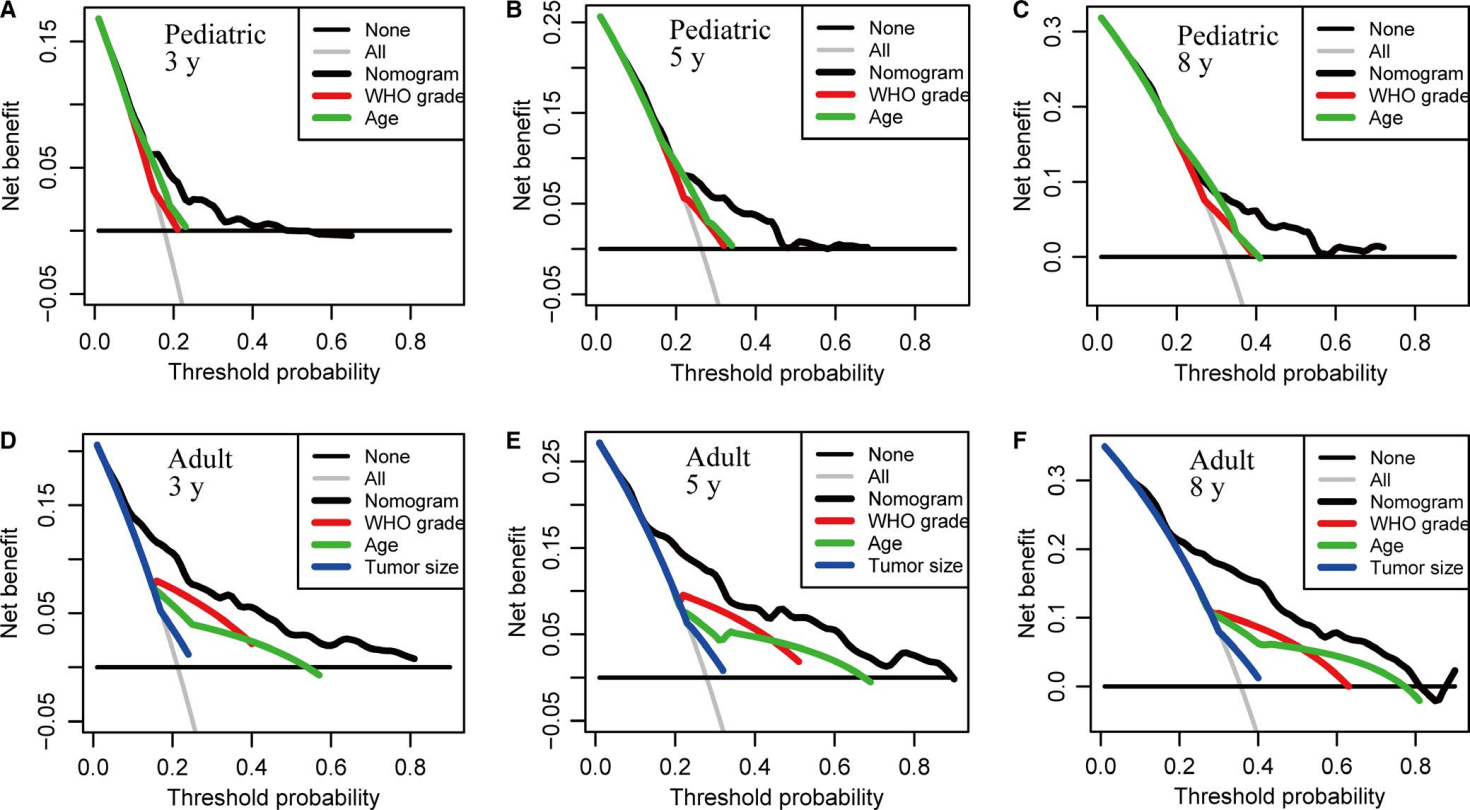
Decision curve analysis for the nomogram model and different conventional clinical characteristics in prediction of prognosis at 3‐y (A), 5‐y (B) and 8‐y (C) point in the pediatric training set, and at 3‐y (D), 5‐y (E) and 8‐y (F) point in the adult training set. The nomograms obtain more net benefits than all other single variables with a wider range of threshold probabilities

We further compared the predictive accuracy between the nomogram models and different conventional clinical characteristics. Comparing the AUCs for ROC curves indicated that nomogram models had best discriminative ability in both pediatric and adult cohorts (Figure 4A‐F). And using time‐dependent C‐index, nomogram models outperformed all other single variables at different times after diagnosis (Figure 4G, H). Finally, DCA demonstrated that nomogram models exhibited the best net benefit for 3‐, 5‐, and 8‐year OS, indicating the favorable clinical utility of these predictive models (Figure 5). In sum, these findings suggested that the age‐specific nomograms had better performance in predicting short‐term or long‐term OS in malignant EPN patients than individual prognostic factors.

### 3.4 Risk classification system

**Figure 6:**
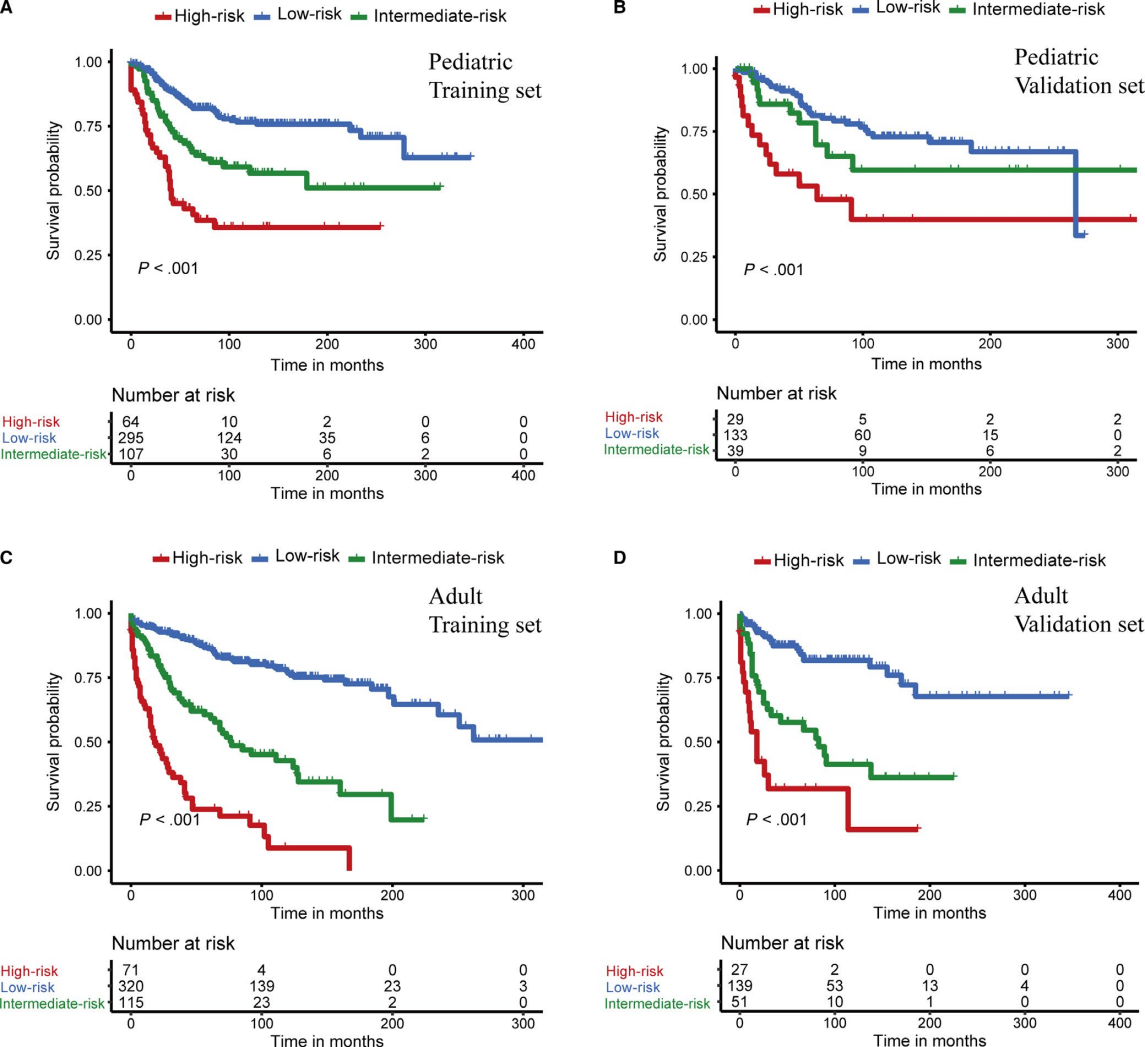
Kaplan‐Meier curves of overall survival for the low‐, intermediate‐, and high‐risk patients in the pediatric training set (A), pediatric validation set (B), adult training set (C) and adult validation set (D)

In addition to nomogram models, risk classification systems were constructed to assign patients into a high‐, intermediate‐ or low‐risk group according to the total score of each patient generated by the nomograms (Table 4). Based on the best cutoff values of corresponding cohorts obtained by X‐tile program, pediatric and adult patients were respectively divided into the low‐risk (score 0‐134 for pediatric and score 0‐90 for adult), intermediate‐risk (score 135‐166 for pediatric and 91‐129 for adult), and high‐risk groups (score ≥167 for pediatric and score ≥130 for adult). The Kaplan‐Meier curves revealed that prognosis of different risk groups could be accurately distinguished by the risk classification systems (Figure 6).

**Table 4:** Score assignment and risk stratification

Variable	Category	Score	
		Pediatric	Adult
Age	≤2	68	
	3‐7	58	
	8‐17	0	
	18‐53		0
	54‐68		43
	>68		100
Sex	Female	0	
	Male	34	
WHO grade	Grade II	0	0
	Grade III	43	68
Tumor size(mm)	<37		0
	≥37		26
Surgery	No surgery	100	41
	Biopsy/STR	43	29
	GTR	0	0
Radiotherapy	No	32	
	Yes	0	
Marital status	Single		21
	Married		0
	Unknown		10
Risk classification	Low‐risk	0‐134	0‐90
	Intermediate‐risk	135‐166	91‐129
	High‐risk	≥167	≥130

Abbreviations: GTR, gross total resection; STR, subtotal resection.

## 4 DISCUSSION

A prognostic model with the ability to predict patient life expectancy is essential in personalizing therapy for tumor sufferers. As a statistical tool being widely applied in the field of cancer, the nomogram calculates all the cumulative effects of integrated key variables to estimate survival via an accessible, straightforward approach.^13^ The present study is, to our knowledge, the first study to develop nomograms for estimation of 3‐, 5‐, and 8‐year OS in EPNs, highlighting the relative contribution of easily accessible clinicopathologic variables to outcome prediction. We have demonstrated excellent discrimination and calibration of these predictive models in both training and validation cohorts. Besides, our nomogram models showed superiority in the clinical utility as well via DCA. Moreover, the novel proposed nomogram‐based risk classification systems could also help neurosurgeons to classify risk more objectively.

Our study highlighted the considerable heterogeneity in prognostic factors between children and adult with intracranial grade II/III EPN. For both pediatric and adult patients, age, tumor grade, and surgery treatment were independent predictors of OS, consistent with previous reports.^10^, ^14^ And we found surgical intervention was the strongest factor influencing the outcome in children, whereas age was the most influential one in adult. Male sex was significantly associated with poorer prognosis in children, while similar association was not found in adult. Based on a study combined all ages together for analysis, Rodríguez et al^14^ also found male sex was a significant predictor of worse survival. However, underlying differences between age groups were unrevealed in their research. Although the tendency of having discrete, pushing borders limits the study on the role of tumor size to some extent for such tumors,^15^ the effect of tumor space occupation cannot be ignored. In our investigation, larger tumor size was an independent risk factor for adult, but not for children. Furthermore, histology has also been found to have different prognostic significance depending on the tumor's anatomic location and the patient's age.^3^, ^16^ Raghunathan et al^3^ reported that the clinical relevance of specific histological features in EPN was associated with the anatomic site of origin. In our study, statistically significant differences between children and adults for tumor grade distribution was observed. These histopathological heterogeneities could also lead to differences in clinical behavior, response to therapy and patient outcomes. Future studies are needed to clarify the age‐specific significance of these histological features.

Significant correlations of marital status with cancer incidence, stage, treatment options, and prognosis have been determined,^17^, ^18^, ^19^ indicating the importance of social support for cancer detection, treatment, and survival. Consistently, we found that unmarried adult patients with EPN were at higher risk of worse outcomes for the first time.

Surgery and radiotherapy are the cornerstones of care for EPN in children.^10^, ^20^ GTR and adjuvant radiation significantly improved the survival of pediatric patients with intracranial grade II/III EPN in our study. For adult, surgery is regarded as the most crucial component of standard treatment,^10^, ^21^, ^22^ in agreement with our results. Remarkably, no clear correlation of radiation treatment with OS was observed. It was reported that radiation treatment should be applied for adult patients with grade III EPN and for adult patients with grade II EPN after STR.^10^, ^23^, ^24^ Grade II tumors taking up the majority in our study might mask the effect of radiotherapy. And these results also suggested that further evaluation on the role of radiotherapy is needed to determine the concrete application condition for adult patients. Furthermore, in spite of extensive investigation and research, the role of chemotherapy in the management of EPN remains unclear. Consistent with our results, several study cohorts of pediatric or adult patients in which the effect of chemotherapy was retrospectively analyzed also failed to demonstrate a survival advantage.^15^, ^25^, ^26^, ^27^ Nevertheless, this modality of treatment might be beneficial to some subsets of EPN patients with the ongoing therapeutic challenges.

Considering the difference between children and adult with EPN mentioned above, rather than simply adjusting for age, the age‐specific approach should be employed to estimate survival for these patients. Furthermore, with the development of tumor molecular biology, a number of immunohistochemical and genomic markers related to the prognosis have emerged, like chromosome 1q status, YAP1 fusion, RELA fusion and so on.^1^, ^2^, ^28^ Due to the limitation of SEER database without genomic data, we failed to incorporate additional variables to improve our models. But it is undeniable that, using easily measurable clinical parameters, our nomogram models are still in possession of favorable discrimination and clinical application value. Also, our study laid the foundation for the establishment of survival estimation model in intracranial EPNs.

Some other limitations should be acknowledged in our study. First, due to its retrospective nature, potentially generating selection bias is inevitable.^29^ Second, patients with missing information on the collected variables were excluded which could lead to a selection bias. Third, misclassification of histological type in EPN seems to be a problem even for EPN studies with central pathologic review.^30^, ^31^ Unable to re‐examine the tumor histology type could raise concern about the accuracy of the diagnosis in our investigation. Forth, SEER database does not include detail information of radiation treatment and chemotherapy. And information on recurrence status, clinical symptoms, comorbidities, and neurological status ware also unavailable. Finally, because our models only took the independent prognostic factors into consideration, survival rate of EPN patients would be underestimated by the nomogram models when it was low. Moreover, although models were established using large cohorts, external validations are still necessary.

## 5 CONCLUSION

Considerable heterogeneity in prognostic factors was highlighted between children and adult in our study. And we constructed and internally validated the first nomograms for accurate estimation of 3‐, 5‐, and 8‐year OS in patients with intracranial grade II/III EPN at an individualized level. The novel proposed nomogram‐based risk classification systems can be also used to facilitate risk stratification in EPNs for optimization of clinical management.

## CONFLICT OF INTEREST

The authors have declared that no competing interest exists.

## AUTHOR CONTRIBUTIONS

Conception and design: Xiangyang Deng. Acquisition of data: Xiangyang Deng. Analysis and interpretation of data: Xiangyang Deng, Dongdong Lin and Xiaojia Zhang. Drafting the article: Xiangyang Deng. Critically revising the article: all authors. Reviewed submitted version of manuscript: all authors. Approved the final version of the manuscript on behalf of all authors: Jian Lin. Study supervision: Jian Lin and Nu Zhang.

## Data Availability

The data that support the findings of this study are openly available in SEER database at ://seer.cancer.gov/data/.
