# Supplementary figures and images for: Personalizing age‐specific survival prediction and risk stratification in intracranial grade II/III ependymoma
Source: Cancer Med. 2019 Dec 3;9(2):615–25. doi: 10.1002/cam4.2753 (PMC6970043; doi:10.1002/cam4.2753)

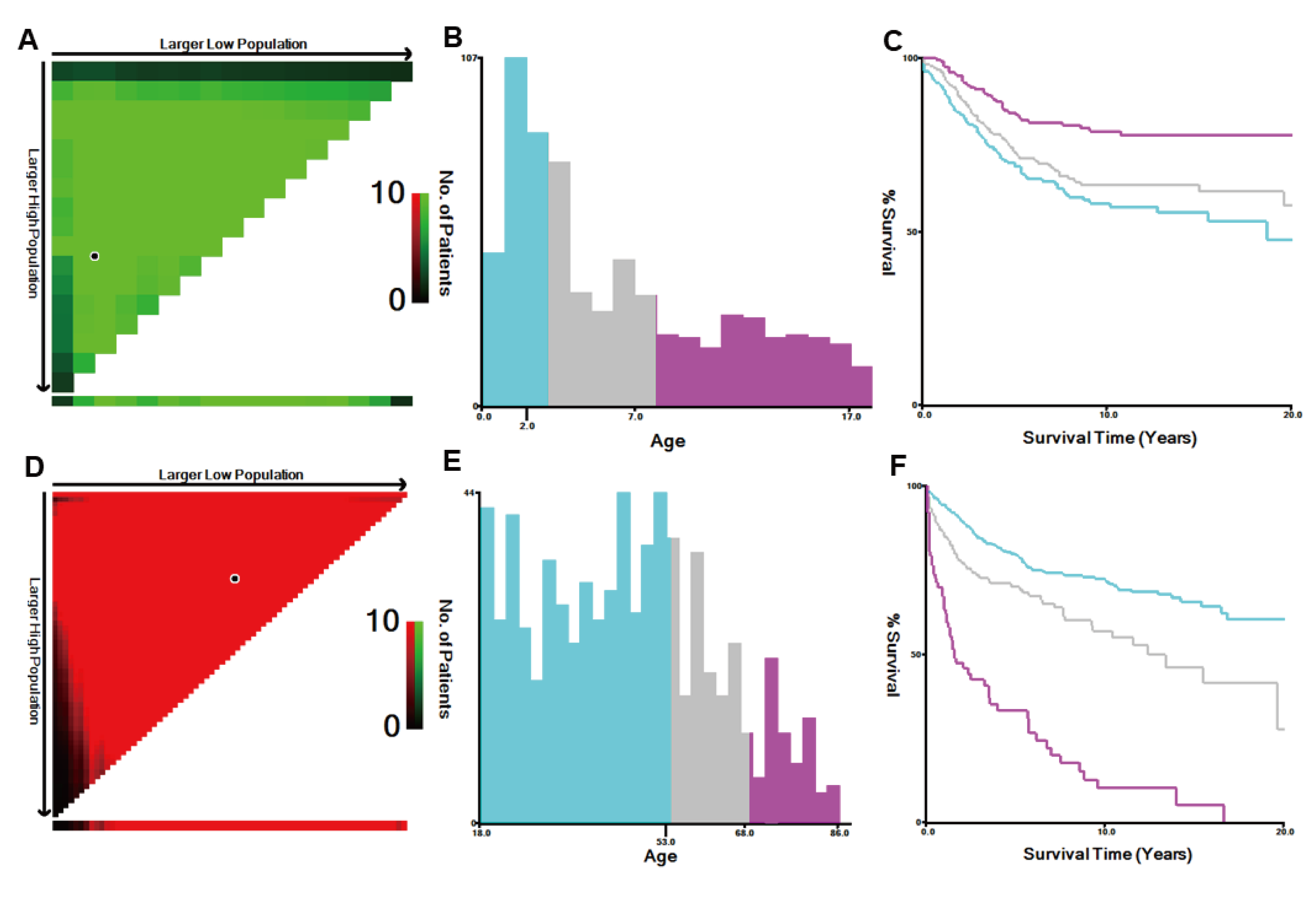

Supplement: Supplementary file 1 [file CAM4-9-615-s001.tif]
